# Cholera Infantum—Treatment

**Published:** 1876-10

**Authors:** B. D. Keator


### CHOLERA INFANTUM—TREATMENT.

BY B. D. KEATOR, M. D.

As preliminary to touching upon the treatment of this disease, I would not fail to mention the flannel bandage, to be worn about the abdomen as a means of prevention; and to protest against the weaning of children (except through necessity) during the season of the year when this disease prevails. In a case of real cholera infantum, in which there are both vomiting and purging, it has always appeared to me that the vomiting did more immediate harm than the purging. It appears to be, in some way, directly connected with the development of cerebral difficulties, and hence our first aim should be to allay the gastric irritation. For this purpose, small doses of hydragyrum submuriate, triturated with white sugar and placed upon the tongue, often prove effectual. Some prefer to add a little plumb, accetate, and both are used with considerable success The cerium oxalate, in doses of one-sixth to one-fourth of a grain repeated at short intervals, I regard quite as successful, if not more so. But the best remedy of all, in my opinion, is creasote or carbolic acid with an alkali. Add to a goblet of water two or three drops of creasote and a pinch of sodium bicarbonate. Of this give a teaspoonful often as required, withhold all drink for a time, place a sinapism, weakened with flour, over the stomach, and you will generally succeed. Or, in the place of this, a drop of creasote, added to an ounce and a half of lime water, and given in teaspoonful doses, will answer as well.

If there is evidence of torpidity of the liver, half to a grain of hydragyrum submuriate, guarded by as much Dover's powder, may be given once in six or eight hours, until the function of that organ be restored. I say this *may* be done, and I have often done it; but of late I have a better remedy. Whether there be dscomposition of bile in this disease, as supposed by a writer before quoted (Mann) or not, I cannot say; but I can certify that the remedy he proposes is an excellent one, almost specific. Here is the formula.

| | | |
|---|---|---|
| R. Aloes socot pulv., | } aa oz i | |
| Potassii sulphat., | | |
| Sodi bicarbonate., | | |
| Caryophyl. pulv., | | oz ss |

Mix, and divide in twelve powders. For use, put one of the powders in a cup, and add three tablespoonfuls of hot water. Dose, one teaspoonful, warm as it can be taken, every half hour, until bright yellow bile appears in the stools.

This is the original, with directions; but I use it somewhat differently. I keep it on hand in quantity, using it both for adults and children for all bilious symptoms. In cases of Cholera Infantum, put a teaspoonful of the powder and a tablespoonful of augar in a teacup, and fill with boiling water. When cool enough to use, give one teaspoonful every fifteen or twenty minutes, withholding drink until vomiting ceases, then every hour until it acts on the bowels as before mentioned. Though having the appearance of a nauseous dose, I find this to remain on the stomach when scarcely anything else will.

But to return. After the vomiting is checked, our attention should next be given tc the diarrhœa. The practice of giving astringents and opiates, with the intention of suddenly checking the diarrhœa, is, I am satisfied from experience, decidedly harmful not to say dangerous.

Opium, or even morphia may be given in most cases with benefit, but in such minute doses as to secure and keep up, as long as required, its soothing and stimulating influence without reaching its stupefying and narcotic effects. If the brain is in a condition of anæmia, what will better tone and send the blood to its empty vessels than stimulating, not depressing, doses of opium?

The effect of subnitrate of bismuth, combined with small doses of Dover's powder—say three to five grains of the former, and one-fourth to one grain of the latter—is sometimes very satisfactory. It appears to exert a soothing and quieting influence on the irritated mucous membrane of the stomach, and thus diminishes general nervous excitement. A convenient form of administration is to add it to the ordinary chalk mixture, in the proportion of thirty grains to the ounce of chalk mixture, with the addition of paregoric in some cases. This, according to the old adage, must be "shaken before taken."

The only astringent allowable in the early stages of Cholera Infantum proper, in my opinion, is zinc oxide. This, besides its astringency, is a nervine, and like the bromides lessens cerebral hyperæmia, and is, on the whole, one of the best remedies we possess for this disease. Not only will it quiet general nervous irritability, but, like the bismuth subnitrate, it has a soothing influence directly on the irritated gastro-intestinal mucous membrane. There may be some who will take exceptions at this, but such is my opinion after an extensive use of it. To neutralize acidity, as well as prevent the possible formation of zinc salts, in the stomach, it should be combined, when used in this disease, with sodium bicarbonate, in about the proportion of eight grains of the sodium salt to each drachm of the zinc.

The action of creasote and carbolic acid are nearly the same, but what difference there is appears to be in favor of the creasote in this disease. The beneficial effect of these medicines is not limited to the arrest of vomiting; but they appear to be among the best means of checking the diarrhœa also, and from their antiseptic properties of preventing fermentation in the stomach, as well as tending to lessen the destructive changes likely to occur in the mucous membrane. In using carbolic acid, the following formula from Professor Davis, of Chicago, is a very excellent one:

R. Crystal carbol acid,...... ................grs.iii
Glycerine (Bower's,)......................oz. ss
Paregoric......................... ..........oz. i
Water........ ....... .......................oz. iss

Mix. Dose, twenty drops every half hour, until the vomiting ceases; then every two hours. (*Vide* Half-Yearly Compendium, January, 1873.)

A favorite formula with me for administering creasote and morphia in proper doses is as follows:

R. Creasote,..... ............... .........gtt. xvi
Morphia sulph.,.......................gr. ii

Ess. peppermint,..........................dr. i
Syr. rhei. aromat.,........................dr. x
Glycerine (Bower's).....................dr. x

Mix. To a child of one year, give from ten to fifteen drops, in a teaspoonful of milk, every two hours while awake. If in the vomiting stage, only a few drops frequently repeated, with lime water and milk, withholding drink.

Much benefit is often obtained in this disease by the use of mucilages; especially should they be given during the use of creasote or carbolic acid. Among these I prefer salep, twenty grains to four ounces of hot water; and next gum arabic.

After the first stages of the disease have passed by, the discharges often become less frequent but more copious, the disease passing into a chronic stage. Here the treatment must be tonic and supporting. Now, with the exception of iron, in cases where it is plainly indicated, no tonic can come in competition with salicin, given in half to grain doses to a child one year old, three or four times daily. It appears to be not only better than quinia, in restoring appetite and aiding digestion, but is the best remedy we have in moderating the diarrhœa, which it does in a gradual and apparently natural way, without the development of the complications following its arrest by ordinary astringent treatment.

We meet with cases of this disease, in its chronic form, which baffles all our skill to arrest, and our only hope is to bridge them over till the approach of cool weather. Such cases I would trust to good nourishment, port wine, and salicin. Whenever the discharges become purulent, or there is any evidence of intestinal ulceration, I have found potassium chlorate, combined with a little Dover's powder, worthy of much confidence.

In those cases which supervene on retrocession of heat rash *lichen tropicus* of Dr. McBride, and which I am confident constitute a large percentage of the worst cases we meet with every season—our aim should be to determine the blood to the surface, recall the rash if possible, and so relieve the congested mucous membrane of the stomach and bowels. For this purpose no single remedy equals belladona. Dr. McBride gives it in half drop doses of the fluid extract, until the cuticular circulation is re-established. This dose was for a child of one year. I would advise, in addition, that the child have a warm mustard bath, or be enveloped in a shawl or other woolen piece, wrung out of warm mustard water, and cooling drink be given. In such cases, we often find the mucous membrane of the mouth very red and hot, and the child ready to swallow anything in a liquid form, be it even a nauseous dose of medicine, for the sake of drink. In these cases, nothing appears to give so immediate relief as scraped ice, in half teaspoonful doses, or in the form of ice cream.

As it has been shown that the brain has been found, post mortem, in opposite conditions, the treatment should, so far as possible, be adapted to the condition present in each case. If there be evidence of cerebral hyperæmia, the best remedies, according to my observation, are the bromides and zinc oxide. The bromides must be given, in such cases, in sufficient doses to control spasm and compel sleep. I have had recoveries from apparently hopeless cases of this character, by keeping the patients asleep by the use of potassium bromide, for several days in succession, only disturbing them at proper intervals for the administration of nourishment in the shape of milk or sweet cream. We thus give the oppressed brain the rest it needs for recuperation. Warm foot-baths, and frequent bathing of the head with cool water, are also proper additional means.

On the contrary, as is more often the case, if the brain give evidences of anæmia, belladonna, from its action on the extreme capillary circulation, and nux vomica, from its power to arouse nerve energy, are the most reliable means at our command. Although opium and belladonna are antagonistic, in some respects, yet it appears to me that when alternated in the proper dose of each, they have been co-workers for good in cerebral anæmia. The head should not be elevated, and should be kept at a warm, even temperature by a woolen cap. These means should be aided by nourishment in the shape of brandy and cream, or pounded raw beefsteak and wine.—*American Practitioner.*

